# Targeting glial scar formation for spinal cord injury: mechanisms, strategies, and research progress review

**DOI:** 10.3389/fnins.2026.1814964

**Published:** 2026-03-30

**Authors:** Chaochao Zhang, Jiaxiao Shi

**Affiliations:** 1 Department of Spine, Cangzhou Integrated Traditional Chinese and Western Medicine Hospital, Cangzhou, China; 2 Hebei Key Laboratory of Integrated Traditional and Western Medicine in Osteoarthrosis Research, Cangzhou, China

**Keywords:** astrocytes, glial scar, inflammatory response, molecular mechanism, neural regeneration, spinal cord injury, stem cells, treatment approaches

## Abstract

Spinal cord injury is often followed by the formation of a glial scar which acts as a major barrier for axonal regrowth and recovery after spinal injuries. The glial scar consists of a complex mixture of cells and extracellular molecules that have different effects; they protect injured areas from further damage but at the same time inhibit axonal sprouting. We have performed a thorough and detailed study of the underlying biological mechanisms in scarring by focusing on the interactions among the astrocytes, microglial, fibroblastic, or immune-related components. With recent advances in experimental model systems and translational studies, here we review therapeutic interventions aimed to modulate scarring, including gene transfer, cell delivery, immunomodulation, and biomaterial scaffolds. Integrating recent advances, the present review aims at providing an effective theoretical basis as well as new treatment approaches for the treatment of SCI patients.

## 1 Introduction

Spinal cord injury (SCI) is a serious disease that causes great loss of sensory and motor function, significantly lowering the quality of life for patients, and the cascade of events after SCI is complicated, wherein the first physical injury leads to secondary pathological events. These are neuroinflammation (neural tissue inflammation), apoptosis (programmed neuronal cell death) and gliosis (scar formation by glia), which create structural and chemical barriers to axonal regeneration and functional recovery (Tran et al., 2022). Our current understanding of these secondary injury mechanisms has been largely derived from preclinical research, underscoring the vital role of animal models in developing and testing potential surgical implants and other therapeutic interventions (Choudhary, 2025). Scar tissue is predominantly composed of activated glial (astrocyte) cells and extracellular components that act both as a barrier in response to injury but also inhibit endogenous mechanisms required for regeneration (He et al., 2024). Recent advances that increase our understanding of the cellular and molecular mechanisms involved in gliosis provide new therapeutic approaches with potential benefits for recovery following SCI.

Formation of glial scars is an essential pathological response following SCI as it serves to limit lesion size, and protect surrounding tissues (Zha et al., 2025). But at the same time pose considerable challenges to axon regrowth. After injury, reactive astrocytes proliferate and migrate into the lesioned site where they secrete inhibitory molecules such as chondroitin sulphate proteoglycans (CSPGs), which impede axonal growth. It has been shown that CSPGs play a key role in creating an inhibitory environment for axon growth, making them the target of SCI rehabilitation therapies. It is clear from this dual nature, protective but also obstructive, of the glial scar that there is a need for more refined approaches to treat the glial scar in such a way as to modulate its formation without interfering with the regeneration of the damaged nervous system.

Recent research has shown that various therapeutic approaches for inhibiting the formation of glial scars, as well as promoting axonal regeneration can be successful (Perez-Gianmarco and Kukley, 2023). For example, an enzymatic therapy using chondroitinase ABC (ChABC) degrades CSPGs, which showed promising outcomes from pre-clinical trials with the regeneration of axons, as well as functional recovery both for motor and sensory activity. The second focus area to target will be modulation of immunity via thoughtful manipulation of the activity of microglia and astrocytes. Scientists are also exploring new ways of creating the ideal environment in which nerves can regenerate and restore their function. In this context, stem cell-based therapies have emerged as a particularly promising approach. Advancements in understanding their mechanisms of action in animal models of SCI are paving the way for potential clinical applications aimed at replacing lost cells and modulating the inhibitory environment (Abusalah et al., 2024; Mili and Choudhary, 2024).

With advances such as single-cell transcriptomics or high-resolution mapping of gene expression within tissues, we are now gaining insight into the heterogeneous cell population and signaling pathways involved in gliosis. The combination of these powerful techniques has allowed us to better define the composition of different glia types and how they interact with each other at a site of SCI, trying alternative therapies. In particular, targeting critical signaling cascades within astrocytes or microglia may be able to switch these cell types from harmful to helpful roles, potentially enhancing the regeneration of damaged neurons following spinal injury. Furthermore, the field is being transformed by the integration of artificial intelligence (AI). AI-driven analysis of complex datasets, including those from diagnostic imaging, is enhancing our ability to characterize injury severity and predict outcomes, which is crucial for designing and assessing personalized therapeutic strategies (Choudhary et al., 2025; Choudhary, 2026).

Biomaterials and tissue engineering approaches are relatively new areas of research for SCI treatment aiming to provide a supportive microenvironment which supports cell survival and differentiation and facilitates axon regeneration across the scar (Sun et al., 2024). In addition, many synthetic biomaterials such as hydrogels, scaffolds or nanoparticles are developed with aims of delivering drugs directly into injured sites for better efficacy and less off-target toxicity (McKenna et al., 2022). Such advances represent a major progress toward overcoming limitations caused by scarring and secondary loss following SCI.

In conclusion, while the development of a glial scar is a barrier to recovery after SCI it can also offer avenues through which we may intervene therapeutically. Improved understanding of how responses by glia to injury are determined will no doubt give us insights as to how this outcome might be improved. In conjunction with advances in scientific instrumentation and biologic agents, may radically change the way we treat spinal cord trauma. Further work on the field will be crucial to develop effective strategies promoting axons’ regeneration and therefore improve functionality in patients suffering from this devastating illness.

## 2 Cellular composition and dynamic changes of gelatinous scar formation

### 2.1 Activation and phenotypic transition of astrocytes

After SCI, there are significant changes in the phenotype of astrocytes: they transform from quiescent states to reactive astrogliosis, followed by their differentiation into mature scarring astrocytes (Abusalah et al., 2024). Such a transformation plays an important role in regulating formation and maintenance of glial scars, which may have a pro-neurogenic, anti-neurogenic, or both effects. The activated astrocyte is characterized by increased expression of glial fibrillary acidic protein.

(GFAP) among others markers that reflect the role they play in inflammation and repair processes (Gorina et al., 2022). The activation of astrocytes has a positive and negative impact, but since these cells secrete CSPG molecules in the scars impairing axons regeneration (Gozlan et al., 2024).

Key regulators such as the transcriptional factor Sox9 play a role in determining astrocyte differentiation, and regulating CSPGs, thus determining functional properties of activated astrocytes (Zhang et al., 2022). Balance between deleterious A1 and therapeutic A2 astrocyte phenotypes determines outcome following SCI. Inflammatory mediators stimulate the production of neurotoxic molecules by A1-type astrocytes which exacerbate tissue injury and promote scarring. On the other hand, A2 type astrocytes produce anti-inflammatory cytokines which promote healing of the nervous system and repair (Chu et al., 2023).

The intricate relationships between astrocytes and other surrounding glia (notably the microglia) further complicate SCI reactions; for example, it has been shown that the behavior and functional state of activated astrocytes is strongly influenced by the microglia via several mechanisms, with transforming growth factor-beta and nuclear factor-kappa B pathways playing an especially crucial role in driving astrocytes toward the A1 state (Meron Asher and Goshen, 2025). Intriguingly, however, these signals are not one-way; in fact, the astrocyte can also signal back and influence the behavior of microglia. Together they form a positive feedback loop which is beneficial for neurons or detrimental through inflammation, with results highly dependent on the microenvironmental conditions found in the injury location (Feng et al., 2024).

A rapidly growing number of papers suggest new treatment modalities which attempt to manipulate the properties of astrocytes so as to drive it into its neuroprotective A2 state (He et al., 2024). For instance, certain drugs were found to including Rho kinase inhibitors are capable of converting A1 astrocytes to A2 astrocytes and thereby enhancing neurological recovery after SCI. Furthermore, novel treatments using exosomes derived from mesenchymal stem cell (MSCs) that promote the activation of A2 astrocytes are being developed and they use the natural anti-inflammatory properties of these molecules against deleterious effects of reactive astrocytes after central nervous system (CNS) injury (He et al., 2024).

In conclusion, reactive astrocyte phenotypic changes following SCI is an extremely complex process that is affected by many factors both intra- and extra-cellularly. Understanding the mechanism involved in astrocytic reactivity will be important when developing effective therapies aimed at facilitating neuronal repair and functional recovery following SCI. Future studies should focus in understanding the molecular pathways that drive the activation of astrocytes and test new approaches able to modulate correctly astrocyte properties at the injured spinal cord.

### 2.2 The immunoregulatory role of microglia

The microglia are resident CNS immune cells that have important roles during the immune response to SCI. Microglia respond to injury by, these cells quickly transition from quiescence into an activated phenotype with morphological changes as well as the secretion of pro-inflammatory mediators. This type of cell transition is also involved in initiating inflammation, as well as fibrosis formation, which can be beneficial or detrimental for neuronal repair and regeneration. It has been reported that activated microglia recruit other circulating blood-derived inflammatory cells into the injury site, increasing the inflammatory response, aggravating the secondary injury process (Zha et al., 2025). However, on the other hand they also have beneficial effects such as clearance of necrotic cellular material or release of trophic factors to protect and repair neurons. It is therefore clear that there are both positive and negative roles for microglia during SCI.

The microglia can be either pro-disease or neuroprotective depending on how they are activated and in which context (Zheng et al., 2022). An important factor that influences microglial activity is the expression level of programmed cell death protein 1 (PD-1), which has been implicated in regulation of inflammation, as well as modulation of reactive astrogliosis after SCI. The expression of PD-1 by microglia, which also modulates the immune system decreasing proinflammatory cytokines and increasing anti-inflammatory cytokines (Cai et al., 2025). This role has great significance since its expression influences the formation of glial scarring, one of the main barriers for axon regeneration. The balance between inflammatory and noninflammatory pathways, controlled by microglia that has a critical effect on defining the cellular environment and promotes or inhibits functional recovery after SCI (Jian et al., 2024).

Moreover, these microglia are able to change their polarization status from the classical activated state (M1), associated with inflammation, toward an alternatively polarized form of activation (M2), which is characterized by its anti-inflammatory effects; this transition can be influenced among others by time post-injury or regional concentrations of cytokines. Cells in M1 state produce reactive oxygen species and inflammatory factors that damage the tissue. On the other hand, M2 microglia promote repair through the production of anti-inflammatory cytokines (Wahane et al., 2021). This polarization is not static, however, and represents an active spectrum that changes according to the healing requirements of the tissue. It has been recently emphasized that understanding this ephemeral progression through microglia states will be key for designing effective interventions at the right time after SCI (Ren et al., 2025).

In conclusion, the microglia are important regulators of immune response after SCI with their activation state and polarization playing a major role for pathogenesis and recovery from spinal injuries. The interplay between microglial activation, of PD-1expression, and functional phenotypes suggests that they are complex contributors to SCI pathophysiology. Pharmacological treatments that aim at modulating microglia activation, but also inducing an environment conducive for neurogenesis could help improve recovery potential post-SCI. Understanding how microglia drive both inflammation and repair will be key in designing effective treatment strategies (Zeng et al., 2026).

### 2.3 Fibroblast migration and the fibrotic process of scarring

During scar tissue formation, fibroblasts are important cells involved (especially after traumatic SCI). After tissue injury, these cells migrate to the site of damage and secrete collagen, as well as many other structural proteins which make up the extra-cellular matrix. This cell migration can be triggered mainly through several biological pathways (such as the TGF-*β* pathway), and cytokine known to induce fibroblast activity and proliferation (Griffin et al., 2023). Scarring involves more than only deposition of collagens, it includes major alterations in the extra-cellular matrix, processes that are necessary to restore the broken tissue structure. In the case of spinal injury, excessive collagen deposition can lead to hyperplastic scars, which could be a physical barrier preventing axonal regeneration and thus recovery of function (Shi et al., 2024).

Furthermore, the fibroblasts operate not alone but as part of a complex cell–cell interaction network; they interact reciprocally with astrocytes among others, together forming a complex scar tissue microenvironment. It is well established that astrocyte-derived signals significantly influence fibroblasts, especially by inducing their migration and proliferation via secreted growth signals, including cytokines or trophic factors (Zhou et al., 2021). These coordinated interactions are necessary to form a mechanically functional scar but can lead to pathologic scarring if the balance is disrupted. The interplay of fibroblast and astrocyte illustrates a complex cell–cell interaction that leads to scarring, thus highlighting a need for better understanding and engineering of therapies that modulate such signals to favorably alter scar formation in favor of wound healing (desJardins-Park et al., 2020).

In addition to their direct contribution toward scarring, the heterogeneity amongst fibroblasts has also long been thought to play an important role during fibrotic processes. Recently, several different fibroblast populations have been identified with distinct activation profiles and ECM production capabilities, which reflects the asymmetric contribution to scarring by these cells (Ma et al., 2020). Some of these fibroblast populations are more likely to become myofibroblasts, which have enhanced contractility and increased collagen synthesis. Myofibroblast differentiation may be stimulated through physical cues within the ECM, which complicate fibrotic mechanisms (Zhang Z. et al., 2021). Understanding such heterogeneity of fibroblast behavior, as well as the mechanisms that drive it are therefore essential to develop strategies aimed at modulating scar formation and promoting wound healing.

The role of EVs released from fibroblasts and their potential to modulate fibrogenesis has recently been demonstrated. They are small membranous vesicles that contain active molecules able to modify target cell behavior, such as immune cells or other fibroblasts, thereby enabling communication between these different cells in the wound healing microenvironment (Fan and Wang, 2025). These observations suggest that targeting the fibroblast signal transduction pathways could represent a novel therapeutic approach in limiting scarring and enhancing tissue regeneration.

Fibroblasts are central to the process of scar formation by moving, stimulating and communicating with other cells around it, all of which contribute to the end product of tissue repair. Given the complexity of these cell behaviors, further investigation is required in order to understand regulation of fibroblast function and its role in normal or pathological scarring. These observations can be translated into new therapies aimed at limiting pathological scarring while promoting functional recovery following SCI as well as other types of wounds.

### 2.4 Involvement of blood-derived monocytes/macrophages in scar formation

After SCI, many blood-derived monocytes accumulate at the lesion site, wherein they differentiate into macrophages. Macrophages play an important role in orchestrating inflammation response and scarring after injury. The differentiated macrophages secrete many cytokines and growth factors that influence systemic inflammation and contribute to glial scarring. The macrophage can be polarized into one of two main phenotypes, known as the classically activated (M1) or alternatively activated (M2) state, all of which contribute to the healing process and scarring, with the M1 macrophage being more involved in an initial proinflammatory response, while M2 promote tissue regeneration and reduce inflammation. The balance of both opposing macrophage phenotypes is critically important to ensure appropriate healing.

The excessive response of M1 macrophages may worsen the tissue damage, promoting a chronic inflammation that leads to more scar formation. In contrast, a robust M2 macrophage response may enhance tissue repair and perhaps assist regeneration (Kjell and Götz, 2020).

The process of macrophage polarization depends on several factors present in their microenvironment and mainly on cytokines. Interleukin 4 has been shown to be an important factor for inducing M2-polarization, which in turn stimulates ECM production, leading to fibrosis formation (Tamaru et al., 2023). All these processes also contribute significantly to the formation of the glial scar, which is considered one of the most prominent detrimental features of SCI that impedes axon regrowth as well as recovery of function. In fact, depletion of macrophages originating from circulating monocytes has been shown to reduce the extent of fibrous scarring, emphasizing their role in defining structure, composition, and organisation of the ECM of scarring tissue (Huang J. G. et al., 2023; Zhang Y. et al., 2021).

Moreover, macrophage recruitment and polarization change with time post-injury; early monocyte infiltration is important in triggering the inflammation response, whereas later macrophage activities are critical to resolve the inflammation and regenerate tissues. In addition, in an experimental model of SCI with mice, the absence of peripheral monocytes/macrophage induced an increase in the formation of glial scars, suggesting that they play a positive role in the control of wound healing (Sahputra et al., 2022). C-C Motif Chemokine Ligand 2 (CCL2) and other chemotactic agents are essential for the recruitment of monocytes into sites of injury; therefore targeting these pathways may be an effective way in which to modulate macrophages after SCI (Li et al., 2024).

Furthermore, interactions of the monocytes/macrophages in circulation with surrounding cells such as astrocyte and fibroblast are complicated which can drive scar formation or prevent its occurrence, where the output is a function of local tissue state, and signaling molecules. It has been shown, that the crosstalk between macrophages and fibroblasts induces more collagen synthesis as well as ECM remodeling, key features of fibrotic tissue (Li Y. et al., 2020). Nevertheless, long term stimulation by macrophages might lead to chronic inflammation and poor tissue repair, thus underscoring the key role that immune homeostasis plays in successful healing.

Blood-derived monocytes/macrophages contribute to scarring following SCI, and these cells are highly malleable, adopting different functional phenotypes, interacting with each other and with other cells in the injury microenvironment, hence affecting wound healing process. In summary, novel therapies that target macrophage modulation and polarization might be an interesting tool to enhance functional outcome after SCI by limiting glial scar formation. Better insight into the role of these immune cells in the formation of the glial scar will enable to design specific treatment options aiming to promote neuroregeneration and thus finally recover mobility after SCI.

### 2.5 The role of oligodendrocyte precursor cells (OPCs) and NG2 + glial cells

Oligodendrocyte precursor cells (OPCs) or NG2 + cells play a key role during spinal injury response and gliosis. OPCs can differentiate into oligodendrocytes that generate myelin within the CNS but also contribute to the scarring process following spinal injury. Researchers have discovered that if OPCs are turned on following SCI they multiply quickly and secrete a variety of inhibitory molecules that prevent axon regeneration. For example, OPCs upregulate the expression of certain neurotrophic cytokines and ECM components, which creates a poor environment for neuronal regeneration (Liu H. et al., 2021). This dichotomy of OPCs that can be reparative yet inhibitory to regeneration simultaneously is consistent with their multifaceted contribution to SCI pathophysiology and furthermore, the presence of OPCs in a scar might enhance the inhibitory environment for neuronal.

These cells also interact during wound repair with other nearby glia as well as with the extracellular matrix, promoting maturation and strengthening of scarring, which can be found, (e.g., Barriola et al., 2020).

Scar formation is also aided by NG2 + glial cells, which are a subpopulation of OPCs involved in ECM assembly and remodeling. NG2 + glial cells modulate ECM composition, an essential part of securing the area of impact. In addition to providing structural support for cells to adhere onto, the ECM also plays an important role in influencing cell function via other chemical cues. Post SCI, NG2 + glia also impact ECM by secreting CSPGs that promote scarring and block axon regeneration (González et al., 2021). This bidirectional relation of NG2 + glia and the ECM is thus crucial because it decides if a regenerative microenvironment in the injured spinal cord can be established or not. Moreover, that NG2 + cells are able to become astrocytes, as well as many of the other types of glial cell in response to pathology, complicating this interplay of cells in a scar (Zhang X. et al., 2021).

The connection of OPCs with NG2 + glia is even further complicated by evidence that OPCs respond to different signaling molecules, including those outside of the CNS, following SCI. Specifically, there has been evidence for an important regulatory role for the Wnt signaling cascade on OPC activity as well as differentiation into mature oligodendrocytes. Dysfunction of the same pathway can lead to overproliferation of NG2 + glia and reduction of neurons, emphasizing that understanding these cellular interactions may provide opportunities for intervention (He et al., 2023). In addition, activation of NG2 + cells has been shown to alter their interaction with other glial cells such as microglia and astrocytes, which can modulate the immune response as well as wound healing processes (Bell et al., 2020).

In summary, OPCs and NG2 + glia are important players for the formation of a glial scar following SCI as they secrete inhibitors and change the ECM, creating an adverse environment for axon regeneration. A better understanding of their roles and cell–cell interactions may lead to new therapeutic strategies aimed at minimizing the inhibitory effects of glial scarring and facilitating recovery after trauma. Future work should focus on how these cells can be manipulated and modulated in order to create an environment that is favorable for the repair of neuronal connections and recovery of function.

### 2.6 Contribution of other cell types

It is important to identify what cell types make up a glial scar following SCI, so that we can understand the complex processes that occur during the wound-healing response and design therapies for interventions. Specifically, ependymal cells, and the other glias also play a role of support for scar tissue formation. The ependymal cells that line the central canal of the spinal cord are an endogenous source of quiescent stem cells which can be mobilized after injury to induce proliferation of ependymal cells, that later takes part in scar formation. Recent reports show that these activated cells can also differentiate into astrocytes as well other glial cell types, thus influencing not only its morphology but also its functionality in the evolving scar (Fabbiani et al., 2020). Moreover, complex molecular communication mediating the crosstalk among ependymal cells with each other and with other types of glia (e.g., astrocytes or microglia), coordinating inflammation and scarring pathways.

Astrocytes are also able to secrete molecules that attract, activate and stimulate microglia; activated microglia may amplify or reduce scarring, depending on their activation status (Takeda et al., 2024). These kinds of feedback loops illustrate a network of cell–cell communication in response to SCI, suggesting that targeting such cell–cell interactions may represent therapeutic interventions.

In addition to the ependymal cell population, a number of other glial cell types are involved with scarring including oligodendrocyte progenitor cells (OPC) and fibroblasts. OPCs, which are essential for myelination and neural activity but can be transformed by damage into reactive astrocytes, thus getting involved in scar tissue production (Zhang Y. et al., 2021). Fibroblasts derived from the meningial tissue or vessel wall proliferate and increase ECM deposition in scars. The expression of Meflin was reported to be upregulated during spinal injury-induced fibrosis, which emphasizes the role of fibroblasts’ activities in scarring process (Morita et al., 2025). These heterogeneous cell types interact to create a dynamic environment which has a profound effect on scarring and subsequent regenerative capacity of the spinal cord.

The intricate network of signaling among all these different cell types is extremely rich: activated microglia secrete inflammatory mediators like interleukin 6 that activate astrocytes and thus contributing to increased scarring. On the other hand, inflammatory mediators produce an environment which may be less supportive of neuroprotection and regeneration (Yang et al., 2024). The balance between these opposing stimuli is critical for determining outcome during the course of injury, as well as subsequent healing potential. It has been proposed that tipping this scale toward an environment favorable for regeneration by inhibiting destructive inflammatory responses while promoting regenerative ones could be beneficial (Lu et al., 2025).

The contribution of multiple cell types including ependymal cells, astrocytes, microglial cells and fibroblasts to glial scar formation highlights a need for understanding how these different cellular populations communicate with each other and control the injury response within the spinal cord through specific molecular pathways. It may be possible to develop novel therapies which modify such cell–cell relationships in order to provide a potential avenue through which the regeneration of injured spinal cord and subsequent recovery can be achieved. Future studies need to be conducted for a better understanding of how each cell type contributes, as well as its related pathways involved, so that specific drugs can be developed to prevent gliosis and promote functional recovery after SCI.

### 2.7 Spatiotemporal dynamics of glial scar formation

Glial scarring following SCI occurs in a three-phase process that can be divided into early, delayed and chronic phases; acute, sub-acute and chronic. During these phases different cells and cell phenotypes are present which significantly influence the architecture and plasticity of scarring. In the immediate aftermath of an injury (acute phase), there is a rapid influx of immune cells such as microglial cells/macrophage responses toward injured tissue that play important roles in clearing up cellular debris and secrete pro-inflammatory cytokines, thus paving the ground for subsequent cell processes. During the transition from early to intermediate wound stage, activated astroglia become the main actors. These glial cells become hypertrophic and start to express the protein GFAP as part of their response (reactive astrogliosis). The latter is not just a passive response;

In contrast, these cells are becoming activated and moving away from their physiological role as support cells toward an active one. Whether this response is beneficial for tissue recovery or detrimental depends on cell context and the pathways initiated at that time (Mohammed Butt et al., 2023).

In late phases of the injury reaction, the glial scar becomes stabilized and forms an elaborate meshwork of extracellular material largely consisting of proteoglycans that contain chondroitin sulfate, together with many other structural proteins. This is not just a passive scaffold but also controls the function of cells in surrounding areas, creating a hostile environment, which might hinder the regeneration of nerve fibres. It is important to note that this process relies on the changing makeup of cell components as they form the scar.

For instance, persistent reactive astrocytes and accumulation of ECM components can lead to scar formation with enhanced fibrosis, is less permissive to axonal growth (Zhang et al., 2024), and the cell populations present in a scar are heterogeneous, where in some regions there is a high density of immune cells while other regions consist mostly of astroglia cells or fibroblasts. This spatial heterogeneity therefore suggests that a study on regional differences in glial scarring is essential, as this information may reveal potential target areas for interventions aiming at enhanced recovery of function after SCI.

Biological implications for such cell behavior include that during scar maturation, the functionality of the involved scar-forming cells is changing, modulate their capacity to respond to injury, and contribute to regeneration. For example, reactive astrocytes are neuroprotective during the early stage while chronic activation could cause negative outcomes (overproduction of scars, or inhibition of axonal sprouting) (Zha et al., 2025). Timing is important for such changes, the success or failure of therapies aimed at modulating gliotic scarring is dependent upon when they are administered. Thus, early therapeutic intervention that targets specific molecular pathways may provide ideal conditions for healing whereas late treatment strategies may be aimed at reducing scarring rigidity and enhancing neural plasticity (Cao et al., 2022).

In conclusion, glial scarring is not only temporally but also spatially dynamic with cell type transition as well as function shift which are crucial for understanding host response after SCI. Scar formation is characterized by an interplay between inflammation and reactive gliosis, and ECM remodeling, which are all factors that have a large impact upon recovery outcome. Novel therapeutics designed to modulate these pathways may be able to enhance functional recovery after injury through encouraging an environment favorable for repair while curtailing the detrimental effects of astrocytic scarring (Zhai et al., 2025).

### 2.8 Differences and similarities of glial scarring in SCI across different species

The extent to which a spinal injury is followed by the development of glial scarring varies greatly between different species: for example, some fish such as the goldfish *Carassius auratus* and the axolotl *Ambystoma mexicanum* show a very different pattern of gliosis and regenerative potential compared with mammals. Aquatic animals like the goldfish or the axolotl have amazing capacity for spinal cord regeneration due to specific functions of their glial cells. Axolotl, for instance possesses an impressive capacity of regeneration not limited to the restoration of limbs but also involving a full recovery of the spinal cord. They proliferate, differentiate into neurons as well as glia, without forming a fibrous scar (as occurs in mammals). The process relies on certain genes to convert glial progenitors from epithelium-like to mesenchymal.

In most mammals including humans, the healing response is often associated with significant glial scarring characterized by proliferation of reactive astrocytes and deposition of ECM molecules such as CSPGs that are known to inhibit axonal regeneration and create a hostile environment for repair (AbouZekry et al., 2025). However, there are also those that have greater capacity to colonize and repair damages creating a good environment for axonal regeneration (Rezaei et al., 2020).

Their comparison gives us a clue to understand the biological mechanism for forming glial scarring and wound healing, in particular, mammalian glial scars generally block regeneration while in other animals such as goldfish or axolotls, a more flexible glial response is observed, which promote repair of the neuronal network. This different response is due to different intracellular pathways that are activated after injury. In mammalian systems there is often persistent inflammatory signaling for the promotion of scar tissue which results in chronic inflammation as well as further tissue injury (Shortiss et al., 2025). In contrast, inflammation in axolotl is acute with rapid resolution leading to efficient healing of tissues and restoration of functions.

Furthermore, studies using methods that profile gene expression have demonstrated differences between species with respect to how their regenerative genes are expressed. For example, in axolotl, that researchers have found that the genes involved with nerve growth and healing are activated within hours of injury. Conversely, in mammals homologous genes are often expressed at lower levels and/or for a longer time period, which are more prone to form scars rather than restore tissues (Patil et al., 2021). These results suggest that understanding the molecular processes underlying glial behavior in high regeneration capacity species could be useful toward designing therapies aiming at limiting scar tissue formation in mammals.

Differences in gliotic scarring and tissue regeneration that are seen in water-dwelling vertebrates like goldfish or axolotls, compared to mammals; these comparative studies have shown us that the response after SCI is complex and also offer a chance at unique regeneration mechanisms not seen in mammals. Comparative models allow us to study regeneration in non-mammalian animals as well as provide new targets for treatment. These strategies can be aimed at modulating the process of glial scar formation, with a view to facilitate spinal cord repair in human patients. Insights gained from studies of such extraordinary regenerators might open up new avenues toward effective tissue regeneration therapies, showing great promise for improved recovery of persons with spinal cord injuries.

### 2.9 ECM components and their regulation

Inhibiting CSPG Chondroitin sulphate proteoglycan is another group of important inhibitors which inhibit axonal regenerations across scar tissue formed by astrocytes following SCI. They belong to the family of CSPGs and they are mostly found in the ECM especially in CNS. Their binding to cell-surface receptors on neurons activates signal transduction pathways that inhibit growth cone motility, and that create an inhospitable environment to regeneration. Their up-regulation after injury is correlated with the loss of plasticity/regeneration ability. In the SCI animal model, it has been demonstrated experimentally that degradation of CSPG by ChABC facilitates axonal growth and functional recovery, highlighting the fact that they are detrimental to neuronal regeneration (Tran et al., 2020). Moreover, they also these PGs.

ECM molecules CSPGs bind to molecules on the ECM, e.g., hyaluronic acid, which is responsible for the generation of specific PNMs that further constrain plasticity and regeneration, as reported in Štepánková et al. (2023). This complexity of action suggests that targeting of the CSPG deposition process and ECM regulation could be an important strategy to enhance recovery after SCI.

In addition to CSPGs, other ECM components such as collagen, laminin, and fibronectin are involved in scarification and tissue remodeling after injury, with collagen I and IV playing an important part in providing mechanical integrity to scars as well as influencing the mechanics of injured spinal cords. Scar stiffness is due to the deposition of collagen fibres that can hinder axonal growth into the scar tissue (Manesco et al., 2023). laminin supports cell adhesion and process outgrowth thus can contribute to regeneration if it is found within the ECM, fibronectin acts as scaffolding protein for cell adhesion and participates in cell migration during wound healing (Bi et al., 2024). The interaction between all these components of the ECM will be crucial in creating a proper environment suitable for tissue regeneration and repair, with studies showing how modulating the physical characteristics of the ECM using hydrogels may influence fibroblasts.

The extracellular environment could be modified in order to foster axonal regeneration and provide a conducive milieu for neuronal growth (Xiao et al., 2025). Of particular importance are the components of the ECM, matrix metalloproteinases (MMPs), which are involved in remodeling of the peritumoural stroma by degrading various ECM molecules including CSPG’s, collagens and which can have a direct effect on scar formation, as well as degradation of the formed scarring. MMP activity must be tightly regulated as excess activity can result in excessive degradation that exacerbates inflammation, while poor enzymatic activity may hinder the correct matrix remodeling and regeneration process (Dolma and Kumar, 2021). Thus, there is a need to balance the activity of the MMPs with that of their natural inhibitors, the tissue inhibitors of metalloproteinase (TIMPs).is essential to maintain ECM homeostasis and facilitate recovery after SCI.

CSPG, laminin-fibronectin molecules, MMP and ECM play a major role in response to stroke at the spinal cord level. The study of their function and regulation can lead to possible therapeutic approaches for promoting regeneration of nerve fibers and recovery of functions after spinal injuries. In addition, strategies aiming at altering ECM composition and its dynamics could offer new tools with dual action (inhibiting fibrosis while promoting regeneration of the nervous system): as demonstrated in recent work (Mohammed Butt et al., 2023).

### 2.10 Signaling pathways in glial scar formation

The formation of scar tissue after SCI is one of the most important pathophysiological processes which are mediated by several pathways including; Integrin-N-cadherin pathway, HMGB1/NF-κB pathway, Rho/ROCK pathway are involved in cell migration proliferation etc., and ECM production—all important elements for scar formation. In particular, through the Integrin-N-cadherin complex astrocytes adhere and migrate toward lesions forming a transient barricade which first limits inflammation and secondary injury but, this favorable microenvironment later becomes a hostile one preventing axonal regeneration and thus hindering recovery of function (Sami et al., 2020). Meanwhile, HMGB1/NF-κB signaling is activated following injury which results in the production of pro-inflammatory cytokines to exacerbate localized inflammation and facilitate wound healing.

The wound-healing response shows that inflammation plays a complex role in SCI, that play a dual role of being both neuroprotectant and deleterious to repair processes. In fact, it has been demonstrated that although inflammation plays a necessary role in protecting against injury at an early stage, it can then block the neurorepair process (Bhatt et al., 2024). Secondly, the Rho/ROCK pathway is also involved in regulation of cytoskeleton organization within astrocytes which determines cell proliferation as well as mechanical properties of scars. Upon activation of RhoA and ROCK, they improve the contractility and motility of astrocytes that are needed to form a functional scar. Nevertheless, overactivation of these pathways can present a barrier for axon regeneration, and must be tightly regulated if they are to be harnessed (Xie et al., 2020).

Understanding these pathways is important to understand how a glial scar forms, but also where we could target specific therapies in order to treat diseases more precisely. It has been shown recently that it might be possible to influence the PI3K/AKT pathway with drugs, which could change neuroinflammation and thus gliosis (He et al., 2022). In particular for SCI models, manipulating this pathway over time has a specific advantage–activation during the post-acute phase of injury seems to provide neuroprotection against apoptosis, while its inhibition in late stages may reduce scarring formation, and promote axon regeneration (He et al., 2022). A second interesting candidate is represented by the YAP pathway, known to be induced through the action of bFGF through RhoA mediated processes. It has been proposed, therefore, that the inhibition of YAP might be a way to prevent uncontrolled astrocyte proliferation and scarring, while also enhancing the process of neurorepair and thus providing an innovative therapeutic option to enhance recovery following spinal injury (Zhang R. et al., 2025).

Along with this crosstalk between different pathways, it has also been shown that the formation of gliotic scaring is not an easy task but depends on several factors, it has been shown that both NF-κB pathway and JAK/STAT pathway are cross-talked to each other, which means activation of NF-κB in astrocytes induces expression of pro-inflammatory factors; then these cytokines can stimulate JAK/STAT pathway thus promoting scarring as well as inflammation (Ageeva et al., 2024). These observations underline that combination therapies able to modulate simultaneously multiple signaling pathways would be beneficial in order to generate an optimal environment for nerve repair and regeneration.

In conclusion, the cellular and molecular pathways of glial scarring following SCI provide opportunities for treatment as well as challenges. The study of the Integrin-N-cadherin, HMGB1/NF-κB, Rho/ROCK and PI3K/AKT pathways thus provide a target for designing therapies which modulate scarring and promote axonal regeneration. Future studies should focus on understanding the interactions between all three of these pathways in order to find ways to modulate them for promoting tissue repair, and limiting the harmful effects of scarring by glia (Ding and Chen, 2024).

## 3 The dual effects of glial scar formation on recovery from spinal cord injury

### 3.1 The protective role of glial scar

Reactive astrocytic scar formation after CNS injuries has multiple consequences during SCI. On one hand, reactive astrocytes can be neuroprotective by they form an anatomical barrier over damaged areas to isolate the inflammation and oxidation reactions which could have reached surrounding brain tissues. The role of scar formation in protecting uninjured axons and maintaining spinal vascular integrity has been highlighted by showing that scaring can be a good barrier, blocking entry of harmful circulating molecules to the spinal cord’s extracellular space, which is critical in neuroprotection and repair (Patilas et al., 2024). Moreover, the scar tissue secretes a variety of trophic factors as well as ECM elements that contribute toward protecting neurons and facilitating repair; in particular, the astrocytic elements of these scars.

Furthermore, it has been shown that astrocytes are capable of producing IGF-1, which is able to modulate glial scar formation through activation of mTOR signaling, a critical intracellular signal transduction pathway. This stimulation promotes enhancement of positive properties of scarring in acute phase following brain injury, which is the same result obtained in Zheng et al. (2023).

However, there are clear limits on the advantages conferred by glial scarring. While this scar acts as an important initial protective response to prevent further tissue injury, whose persistent accumulation is known to inhibit axon regeneration and impair recovery, at least partially through their secretion of inhibitory molecules into the extra-cellular space (such as CS PGs). This kind of paradoxical effect suggests that the role of a glial scar evolves in time, from an initial beneficial one to a detrimental one at late phases after injury. It is known that these scars may largely impede neural regeneration by blocking the extension of axons which leads to permanent neurological deficits (Shafqat et al., 2023). This evidence highlights the complexity of glial scar biology, therefore underscoring a need for therapies that can modulate scar formation and function in a temporally controlled manner during the healing process.

Furthermore, there is an interplay between the formation of a glial scar and inflammation which determines the composition of the cell population after SCI. Scar-forming reactive astrocytes interact with microglia and other immune cells, thus modulating the inflammatory environment. After SCI, activated microglia release pro-inflammatory mediators which promote secondary injury and scarring, as reported previously (Bhatt et al., 2024). However, some of these modulators such as IL-10 can also modulate inflammation levels and improve the environment for neuroregeneration through modulation of scar formation, according to study (Patilas et al., 2024). Our results indicate that in any future therapy, both fibrosis formation and associated inflammation need to be considered.

Finally, although the development of a glial scar after SCI can be advantageous during the initial stages of disease by creating a physical barrier between injured and uninjured regions, their physiological role is quite complicated and it depends on the situation. In other words, what was once considered a positive trait has become a potential negative one, meaning it is important to find effective treatments able to maximize the advantageous features of this kind of scar without its deleterious effects over axonal regeneration. Understanding how are formed the glial scars as well as the interaction between them and the immune response could be crucial for future therapies against SCIs and improving recovery after injuries.

### 3.2 Inhibitory role of glial scar formation

After SCI, formation of glial scarring presents great challenges to axonal regeneration and functional recovery. Scar tissue, which are mostly made up from activated astrocyte cells form a physical barrier to prevent axons growth over the site of injury. Astrocytes proliferate rapidly and migrate toward the site of damage, producing an ECM rich in extracellular products surrounding the infected tissue. In addition to forming physical barriers, the scar also changes the biochemical environment in a way that suppresses axonal growth. One of the main molecules present in the scar and known to inhibit axon growth are CSPGs, that are shown to inhibit progression of growth cones, which are essential in axonal regeneration. CSPGs bind on cell surface receptors of neurons that initiate pathways inhibiting growth cone motility as well as promoting. These processes increase neuronal cell death and aggravate the functional deficits following SCI (Gao et al., 2022; Li Z. et al., 2020).

Furthermore, secretion of inhibitory molecules by the glial scar poses additional barriers for recovery. Reactive astrocytes, for instance produce numerous signal molecules like cytokins and chemokin that promote an inflamatory environment that in turn induces secondary injury and increases neurodegeneration. All these inhibiting components do not just hinder axonal regeneration, they also prevent reestablishment of vital circuits necessary to restore motor and sensory functions. It has been suggested that approaches aiming to decrease the formation of glial scarring or alter their inhibiting properties could enhance CNS regeneration (Xing et al., 2023; Wang et al., 2022).

In addition to the physical and molecular barriers imposed by a glial scar, there is also an active interaction between neuroglia and immune cells that plays a role in SCI pathology. Immune cells, especially macrophages, enter in the damaged site and modulate astrocyte activity to induce their transformation into scarring state. This communication takes place through several different molecular pathways including the P2Y1 receptor pathway whereby ATP released from macrophages is acting as a chemotactic agent on astrocytes, enabling them to accumulate in the lesion area, leading to scarring later on (Ono et al., 2023a,b).

Recent therapies are designed to manipulate the glial scar environment to encourage neuronal regeneration, and hydrolysis of CSPG’s via administration of ChABC has shown promise as an experimental treatment in rodent models, allowing axons to grow into scar tissue (Khalil et al., 2022). In addition, investigators have explored whether individual microRNAs such as miR-124 could enhance Schwann cell and astrocyte interactions, which can possibly reduce the growth-suppressing characteristics of the scarring (Li Z. et al., 2020).

Finally, the presence of glial scar after SCI contributes to a negative impact on regeneration via several mechanisms, such as structural barriers, cellular communication (molecular) or other complex interactions between cells. Understanding the mechanisms that are involved in this process is vital to develop strategies able to support nervous system regeneration and restore function after injury. A better understanding of how the formation of a glial scar, as well as its inhibitory factors can be controlled may open new avenues in the treatment of patients with SCI (Hai et al., 2023).

### 3.3 Dynamic functional transition of glial scar cells

The dynamic role of elements within a glial scar (i.e., astrocytes and microglia) is an important contributor to post SCI pathophysiology. Following spinal cord injury, astrocytes undergo reactive changes that convert them from a quiescent to an active phenotype characterized by proliferation as well as altered gene expression; this change is temporally regulated and has both positive and negative consequences for recovery. Early on, activated astrocytes are considered to be protective in nature as they form a glial scar which isolates the site of damage from the rest of the brain. On the other hand, the longer these cells are activated the more they produce scar tissue that acts as an obstacle to axon regeneration resulting in poor nervous system recovery (Ewing-Crystal et al., 2024). Furthermore, the use of early intervention strategies to prevent or limit glial cell activation may further enhance the neuroprotective effect exerted by activated astrocytes, while also decreasing their participation in scar formation later on during the healing process (Perez et al., 2021).

As resident immune cells of the CNS, microglia are regarded as first line of CNS defence and have been shown to undergo profound functional alterations post SCI. During the acute stage of injury, these cells get activated, migrated toward the site of injury where it performs its significant role like phagocytosis and secretion of neurotrophic factors. However, persistent activation of microglia could turn toward an activated proinflammatory phenotype with upregulation of inflammatory mediators aggravating tissue injury and leading to formation of glial scars (Liu J. Y. et al., 2021). Microglia-astrocyte interaction is very complex, as activated microglia may modify astrocytes’ activity and therefore affect inflammation and scar formation (Takeda et al., 2024). Understanding of these events will help to develop strategies that modulate the neuroinflammatory response after SCI with a view toward promoting recovery.

Furthermore, plasticity among glial cell populations is important to determine how much function can be regained after SCI: certain subsets of astrocytes are characterized as “stem-like,” which can be induced to become neurons or glia (oligodendrocytes) under the right conditions. This plasticity has a great potential to be exploited therapeutically in order to promote tissue repair and regeneration, as reported in Zhu et al. (2026). Elucidating the underlying molecular pathways of these transitions such as YAP-dependent pathway, is essential to develop targeted therapies that optimize the recovery of function following spinal injury (Pang et al., 2021).

The complicated interactions among different glial cell types (e.g., astrocytes, microglial cells and fibroblast cells), plays an important role in modulating the plasticity of a gliotic scar, where fibroblastic secreted factors, such as a variety of cytokines and trophic factors that modulate activity in astrocytes and microglia to influence tissue repair after injury (Zhang Y. et al., 2021). The complexity of this communication pathway highlights the importance that coordinated reactions between different types of neuronal cells play in successful CNS repair following SCI.

Overall, it is clear that there are dynamic shifts in function of various elements of the glial scar including the astrocytes and microglia, are of great importance in understanding the intricate role they play post-spinal injury, as these cells have neuroprotective effects limiting lesion progression and promoting repair, however, prolonged stimulation could have detrimental effects like scarring which hinders axonal growth. New therapies should be developed in order to modulate such compensatory mechanisms, maximizing beneficial effects and preventing detrimental consequences associated with fibrosis. The study of temporal and phenotypic properties of reactive gliosis is an essential step toward the development of effective recovery strategies after spinal injury.

### 3.4 Interaction between glial scar and inflammatory response

The complex interaction between glial scar and inflammation is a key part of the SCI pathology. After SCI, a rapid inflammatory response which can be essential to initiate the repair process but also can exacerbate the damage as well as induce gliosis. These scars are mostly composed of activated astroglia, glia and other extracellular components. They can have positive or negative impacts since on the one hand they limit inflammation spreading and protect neighboring neural tissues but.

On the other hand, it also sets up a physical and molecular barrier which inhibits axon regeneration as well as functional recovery (Pang et al., 2021). Which of those two effects is dominant seems to be determined by whether or not resident inflammatory cells are activated. In particular, the accumulation of M1-like (proinflammatory) macrophages may worsen neuronal injury and favor fibrotic scaring, while the anti-inflammatory, wound-healing, neurite-outgrowth promoting M2-macrophages are involved in repair mechanisms (Wang et al., 2022). Notably, these patterns of immune cell activation change over time after injury reflecting dynamic nature of inflammation and interactions between immune and non-immune (glia) cells.

The recent work has also demonstrated a crucial impact on the identities and morphologies of glial scarring by immune cells differentiation, especially with regard to nerve regeneration, and it is shown that reactive astrocytes may differentiate into several different functional phenotypes, including two of the most studied types, namely A1 and A2 which have very different consequences in terms of cell survival or glial scarring. The A1 type promotes harmful neuroinflammation while the A2 phenotype exhibits beneficial neuroprotective/tissue-reparative functions (Perez-Gianmarco and Kukley, 2023). Furthermore, microglia, which are the main innate immune cell type in the CNS and can have a profound influence on inflammation and therefore scarring. It has been demonstrated experimentally that the absence of microglia prevents the adequate scarring process with consequent increase of inflammatory response and neuronal degeneration, thus underscoring the critical roles they play in CNS protection after SCI (Zhou et al., 2023). This suggests that interventions designed to modulate microglial activity, as well as promote a switch toward reparative activation states, might enhance outcomes following SCI.

In addition to directing immune cell polarization, the signaling pathways activated during inflammation also contribute significantly toward defining the characteristics of the glial scarring response. For example, it has been reported that both the p38 MAPK pathway and the Akt pathway are involved in regulating inflammation and glial scarring formation (Wang et al., 2022). Indeed, experimental studies have demonstrated that inhibition of such pathways leads to a reduction in scarring as well as improved functional recovery, which may be potential targets of therapeutic interventions following SCI. Communication between glia and the innate immune system involves a variety of mediators, such as cytokines, and chemokines which might promote or inhibit inflammation and fibrosis. Interestingly, IL-10 is an important regulatory cytokine which modulates the immune response and promotes repair by controlling gliosis (Patilas et al., 2024).

The connection of the formation of the glial scar with an inflammation response can be important for outcome after SCI because this process includes various cellular elements, which are involved in the healing of the lesioned area. Understanding the underlying mechanisms could allow developing targeted therapeutic interventions to promote neuronal repair and function. Future studies should focus on defining specific pathways and cell-to-cell interactions modulating the link, and exploring new avenues to manipulate inflammation and fibrosis such that it enables CNS regeneration.

### 3.5 The therapeutic significance of glial scars at different stages

Glial scar formation has a complex relationship to spinal cord injury, depending on the stage of injury or recovery. In the immediate aftermath of injury, the formation of such scarring serves an important neuroprotective role in limiting inflammation, and avoiding further injury (Zhang Y. et al., 2021). At this initial phase, there is increased astrogliosis which forms a scar barrier to prevent axon regeneration. Therefore, therapeutic strategies aimed at modulating the excessive activation of glia cells in that early phase may possibly set up favorable circumstances for neuron survival and regeneration (Tran et al., 2022). Nevertheless, as the wound enters the intermediate and chronic phase where scar tissue becomes a hindrance rather than an aid. In those latter phases there is a reorganization in the scar structure with an increased inhibition for axonal growth as more inhibitory factors are secreted by the scar tissue.

The molecular content of the ECM, such as proteoglycans that contain CSPGs, plays an important role in the process of neural repair (Tamaru et al., 2023). Therefore, therapeutic strategies have to follow different phases; starting with the inhibition of glial cells’ reaction in the initial phase after trauma, then moving toward altering the properties of scar tissues in intermediate and longer term regeneration periods for promoting axonal regrowth. The rationale for this step-wise approach is that it targets various biological barriers, which are overcome in a suitable time point aiming for optimal functional recovery.

Given that glial scarring is a complicated process, it may require multi-stage therapy; in early stages after injury, we need to focus on reducing inflammation as well as minimizing excessive activation of glia cells, perhaps by administering anti-inflammatory drugs or modulators of the glia (Ono et al., 2023b). It has been shown, for example, that MSCs could reduce fibrosis and promote functional neurologic recovery through modulation of the immune response (Clifford et al., 2023). During later phases of rehabilitation, treatment strategies be redirected toward altering scar architecture in a way that reduces the barrier properties of scar tissue for axon regeneration. Promising candidates are molecules which degrade CSPG, or induce astrocytes to become ‘more permissive’ for axon growth (Perez-Gianmarco and Kukley, 2023). Furthermore, including synthetic materials and scaffolds which could bridge over the scar area may provide routes of regeneration to the axons, potentially improved function.

The key question about the role played by scars is how they adapt following trauma (Sullivan et al., 2021). Understanding changes over time within a scar’s cellular content and functionality will be crucial when designing strategies aimed at optimizing recovery after injury, with recent evidence showing that the timing of intervention may play an important part depending on the nature of the injury. Thirdly, we must understand the mechanism by which scarring arises and recovers, and developing combined therapies targeting inflammation as well as ECM repair for enhanced recovery (Takeda et al., 2024). This information will aid in the development of new treatments that retain some positive aspects of scarring, yet reduce negative barriers to axon regeneration. It would be crucial to have such a view in order to develop therapeutic interventions aiming at improving the regeneration of injured spinal cords.

## 4 Therapeutic strategies targeting glial scar formation

### 4.1 Gene regulation and molecular intervention

Gene regulation is central to determining how astrocytes respond following SCI; namely by restricting their activation and subsequent formation of gliotic scar. Chief among these regulators are including Sox9 and Ski, which are key factors in this process; SoX9 is an important factor that maintains astrocyte identity (can control the overstimulation of their own activity, for instance by regulating the expression of inflammatory or proliferative genes). It has been shown that manipulating Sox9 expression dramatically affects the course of reactive gliosis suggesting that controlled manipulation of Sox9 may be an effective therapeutic approach to limit scarring following SCI (Da et al., 2021).

All this occurred concomitant with the augmented formation of gliotic scar tissue suggesting an important role for Ski during reactive astrogliosis after injury (Klatt Shaw et al., 2021).

In recent years, the field of molecular biology has provided new methods to modulate scar-related genes as a strategy for SCI therapy. In particular, via small interfering RNA (siRNA), and by utilizing gene-specific knockout strategies have proven to be very promising toward this goal. It has been shown, for example, that targeting the expression of genes involved in inflammation by silencing them with siRNA could reduce significantly the secretion of pro-inflammatory molecules, thereby reducing the abnormal increase in gliosis that is typically seen after SCI. This has been demonstrated by several research groups, with inhibition of the interleukin-6 (IL-6) gene and tumor necrosis factor-alpha (TNF-α), decreased astrocyte reactivity, and improved neurological outcome (Malvandi et al., 2022). The same observations were reached in parallel by means of a genetic ablation approach, which demonstrated that the absence of certain genes results in an environment that is permissive to axon regeneration. In particular, experimental models in which the CSPG gene was removed, there were fewer scars formed as well as more preserved neural tissue (Zheng et al., 2026).

All these cell-molecular therapies are holistic approaches for treating SCI since they all target crucial pathways with specific molecules, or utilize cutting edge biotechnologies such as scientists are able to modulate the cell response to injury leading potentially to better neurorepair and recovery of movement abilities. Secondly, as more is learned regarding how astrocytes are activated biochemically, new treatment options will arise, offering new avenues toward innovative strategies to promote spinal tissue regeneration and minimize deleterious effects of scar tissue formation. Ongoing research into these physiological mechanisms will be important for developing effective SCI therapies, thus, enhancing the quality of life for patients and their everyday lives (Wang et al., 2023).

### 4.2 Stem cell transplantation therapy

SCI has been an indication for stem-cell based treatment, not only because of the capacity of these cells to reduce inflammation, reduce scarring formation, and improve the healing of nerve tissues. Of all the different kinds of stem cells, MSCs are particularly beneficial to use as a treatment modality. They can also modulate inflammatory processes following injury, which creates a favorable environment for tissue regeneration. It has been shown that they secrete several trophic factors and cytokines with neuroprotective effects on viable neurons as well as stimulating proliferation and differentiation of endogenous neural progenitor cells (Tahmasebi and Barati, 2022). Secondly, MSCs inhibit formation of glial scaring which is one of the major known impediments for axonal regeneration. In fact, glial scar formation represents an important barrier to axon growth after SCI. Transplantation of MSCs at the site of damage reduces scarring and creates a microenvironment favorable for tissue repair (Chen et al., 2023).

Apart from MSCs, Olfactory ensheathing cells (OECs) are also getting attention of researchers as they help in the regeneration of axons after SCI. This is just one example of a special type of glial cell that normally helps axons grow and navigate through the nasal sensory tract, but can also modulate amounts of CSPG molecules – which are part of a barrier-forming process called scarring in the CNS (McKenna et al., 2022). These cells promote axonal outgrowth by modifying the expression of CSPGs (inhibitory molecules blocking nerve growth), thereby improving the possibility for recovery in nerve function from SCI. *In vivo* trials on animals with SCIs showed improved mobility as well as tactile perception when OECs were transplanted into these patients’ bodies (Huang L.Y. et al., 2023). It may be that combining MSC with OEC would have a synergetic effect based on their individual advantages for better outcome of SCI treatment.

Clinical trials to test the use of stem cells as a treatment option in SCI patients are just beginning, with preliminary evidence indicating that they could help improve neurological outcomes as well as the quality of life for patients (Agosti et al., 2024). However, a number of challenges remain, such as determining when to perform a transplant, what cells should be used, or how best to deliver these in order to achieve efficacy without toxicity. Cell therapy combined with bioactive scaffolding material and neurotrophic factor(s) can potentially improve the survival and function of cells, thus improving the efficacy of treatments (Zhang X. et al., 2025). The field of stem-cell therapy in SCI is currently moving very quickly, with ongoing efforts to elucidate underlying mechanistic pathways that mediate therapeutic benefits, and refine protocols in order to facilitate their clinical application.

### 4.3 Pharmacological treatment

Pharmacological interventions in SCI are far from new, whereby treatment now aims to modulate inflammation and promote axon regeneration by various pharmacological agents, including Phenserine is an interesting example of an anti-inflammatory drug with the ability to inhibit aberrant inflammation and excessive scarring at sites of injury. In fact, evidence suggests that it has an important role on modifying the inflammatory milieu toward a permissive one for axonal sprouting, as well as reducing gliotic scar formation (a barrier for axons regeneration and recovery after SCI (Nascimento et al., 2025). Since uncontrolled inflammation at the time of the acute phase of SCI may worsen the injury itself, drugs modulating such an inflammatory response, including the use of Phenserine), represent a logical treatment approach for enhancing recovery potential.

Another drug of interest is Glycyrrhizin that inhibits HMGB1/NF-κB pathway, one of the pathways implicated in inflammation following SCI, which has been shown to reduce glial scar formation and promote an anti-inflammatory environment conducive for axonal growth and repair (Shang et al., 2025). This process is relevant because reactive astrocytes and other glial cells are part of the scar which forms a mechanical barrier as well as a chemical one preventing axon regeneration. By controlling inflammation and limiting gliotic scarring, Glycyrrhizin may increase the likelihood of rehabilitation in patients with spinal cord injury.

Furthermore, modulation of EV release via targeting Rab27a provides potential therapeutic opportunity as it is involved in CSPG excretion, substances known to inhibit axonal growth and play a role in scarring. Researchers have attempted to modulate Rab27a function as well as manipulate CSPG release in order to create an environment conducive to axon regeneration (Sanghvi et al., 2025). In this sense, it is clear that understanding how scarring occurs at a cellular level could allow pharmacological manipulation of such mechanisms.

In conclusion, the existing drugs targeting inflammation or glial scaring are currently used in SCI therapy; these include Phenserine, which is an effective drug against inflammation-induced tissue destruction and a candidate for neurorestoration treatment; and Glycyrrhizin, which is also a promising agent that has shown some encouraging effects in several studies. Recently, novel strategies focusing on Rab27a have been developed to improve SCI recovery. Further research is needed to develop effective pharmaceutical treatments which might significantly improve the lives of people with SCI.

### 4.4 Physical rehabilitation and training interventions

Rehabilitation of SCI individuals by regular physiotherapy may significantly improve the condition, with the main goal of recovering motor functions and improving the quality of life. One of these therapies is Body Weight-Supported Treadmill Training (BWSTT), which has been proven successful and may have great promise for overcoming two critical barriers to neurorepair, namely, increased astrogliosis and formation of glial scar tissue. Research indicates that this form of training can reduce excessive scar formation as well as aid recovery from neurological damage through enhanced motor coordination and locomotor performance after SCI. Specifically, experimental evidence that BWSTT can reduce astrocyte reactivity, which plays an important role in scarring, thus providing an environment conducive to regeneration of the nerve fibres with subsequent re-establishment of function (Zhai et al., 2025). In addition, this treatment modality correlated with better gait features and muscles.

BWSTT has proven to be advantageous in patients with SCI. This treatment is crucial to building muscular power and endurance, key tools to regain mobility abilities and independence among people with SCI.

In addition to the above mentioned benefits in terms of physical well-being, BWSTT also showed promising results regarding psychological aspects and thus contributes to a better quality of life during rehabilitation. As treadmill training is highly structured, it allows for repetitive stepping cycles, which allows the patient to recover physically, while simultaneously building their confidence and motivation due to seeing positive change. This is especially useful since it has been shown that the presence of depression or anxiety can impact on rehabilitation outcome. Therefore, performing BWSTT while rehabilitating may improve both motor function and resolve SCI-related psychological problems (Pisano et al., 2025).

In addition, BWSTT was found to be complementary with other treatment modality such as FES or robot-assisted walking therapy, aiming at enhancing the overall motor function because they are able to cover different aspects of recovery (i.e., muscle contraction), coordination of movement, and ability to perform activities of daily living. In particular, robotic gait orthoses have been reported to complement the effect of BWSTT by providing additional support in posture control while also allowing for higher intensity training periods. Such combination is expected to maximize recovery potential among both mildly and severely affected individuals (Duan et al., 2021).

Incorporation of novel rehabilitative interventions, like BWSTT, to routine clinical practice is a major advancement toward SCI management; these treatments address both physical and psychological recovery. Potentially improve the strength and quality of life in individuals suffering from spinal cord injury. Given that research on the subject is still being conducted further studies should be done to improve upon them so as to make better use of these treatment techniques and provide them to people all over the world.

### 4.5 Biomaterials and tissue engineering

Biomaterials are playing an important role in the area of tissue engineering to treat SCIs. Recently, micro-tissue engineered neural networks (micro-TENNs) have been developed, which can act as scaffolds across injured regions of the spinal cord and provide a scaffold for axon regeneration. Such scaffolds mimic the characteristics of native ECM providing a suitable environment for neuronal survival and growth. It has been demonstrated that such scaffolds can facilitate the specific regeneration of axons as well as reduce detrimental effects of gliosis, a major roadblock in recovery after SCI (Li et al., 2022). Moreover, coupling the above-mentioned scaffolds with bioactive molecules (e.g., neurotrophic agents) was also shown as a way of improving its reparative potential, thus creating an optimal environment to support the regeneration of neural tissue (Ma et al., 2025).

Scaffold material choice is also important for avoiding negative glial responses which could inhibit axonal growth, as scaffolds can be tailored to have specific properties (mechanical, chemical) to provide structural support while modulating the surrounding immune response. Polymers such as hydrogels are able to be tailored in order to release drugs slowly, that are able to specifically target the inflammatory milieu (Zhu et al., 2025). Moreover, their mechanical properties can be tuned to mimic those of native SCs, a key parameter to promote cellular migration and axon outgrowth (Zheng et al., 2025).

Moreover, developments on 3D bioprinting technologies allowed to produce complex scaffolds aiming at maximizing the cell adhesion, multiplication and specialization. Such artificial scaffolds may be equipped with guiding channels, gradients etc., which guide migrating neurons and promote growth cones to form connections at defined positions, thus, offering a better chance for recovery from neurological deficits after SCI (Jiu et al., 2024). The inclusion of electrically conductive materials in such scaffolds is promising as it can aid in neuronal electrical communication and enhance the overall neural regeneration process (Qin et al., 2023).

In summary, application of biomaterials in SCI therapy is an active and rapidly expanding field that has great promise for improving clinical outcomes by providing mechanical support,reducing inflammation of supporting cells (glia), and delivering drugs that promote repair, significantly enhancing recovery after SCI. As science continues to advance further, integration of new biomaterials and next-generation TE strategies will likely produce improved therapies for those affected by SCI, with the long term aim to restore mobility and tactile perception for patients suffering from this kind of injury (Ralph et al., 2024).

### 4.6 Immunomodulatory strategies

For the therapy of SCI, most immunomodulatory strategies target mainly macrophage polarization, which could promote anti-inflammatory M2 polarization and create a better microenvironment for scaring repair. After SCI, macrophages play an important role in regulating inflammation, who have a significant influence on tissue repair through their activation states, as pro-inflammatory (M1) macrophages aggravate neuronal injury, whereas M2 macrophages support tissue repair. Recently, it is reported that inducing a switch in polarization of macrophages to an anti-inflammatory (M2) type reduces inflammatory injury and enhances functional recovery after brain injury. Such as, microglial exosomes containing anti-inflammatory agents that shift macrophage polarization toward the M2 phenotype; reduction of oxidative stress and inhibition of glial scarring, a major obstacle to neural repair.

Axonal regeneration after an injury is possible by applying certain treatment strategies (Cui et al., 2025). Some novel therapeutic methods based on biocompatible materials, especially those containing immunomodulating molecules in a hydrogel structure were designed for creating ideal environment which promotes M2 polarization of macrophages and neurorestoration. These smart hydrogels may then respond to this acidic environment in a wound brain, slowly secreting key cytokines like IL-4 involved in driving toward an M2 phenotype (Xi et al., 2020). By modulating macrophages with the aim of controlling inflammation, minimizing additional tissue damage and, at the same time, enhancing the spinal cord’s endogenous healing capacity.

In addition to promoting the polarization toward M2 type, decreasing pro-inflammatory cytokine release is also critical for downregulating the neuro-inflammation response and limiting secondary neuronal loss following SCI. Following SCI-induced inflammation, numerous cytokines are released from injured sites which exacerbate tissue necrosis and hinder recovery. Therefore, the treatment of these mediators is crucial. Moreover, it has been shown that an electrospun scaffold able to deliver IL-10 in a localised fashion (at the injury epicentre) could help control inflammation both acutely and chronically following SCI (Sun et al., 2024). Such a local delivery strategy has two major benefits, avoiding systemic toxic side effects associated to most common immunosuppressants and enhancing their efficacy by limiting the anti-inflammatory response to the desired area. Moreover, intentional modulation of specific immune pathways such as NF-κB inhibition by pharmacological agents significantly decreased pro-inflammatory cytokine production, thereby improving the outcome of functional recovery (Ren et al., 2025).

The combination therapy which involves promotion of M2 polarization while inhibiting proinflammatory cytokines secretion is an ideal strategy in modulating the post SCI inflammatory responses, since it can deal with both the immediate detrimental effects brought about by the injury as well as provide a conducive environment for regeneration and recovery. As further studies might lead to the application of such immunomodulatory therapies among SCI individuals, leading to better results, with a higher quality of life.

### 4.7 Targeted therapy of signaling pathways

The complex molecular pathways associated with the pathophysiology of SCI represent a huge opportunity for developing therapeutics that target glial scar formation and improve regeneration outcomes. Of those pathways, the second most significant path is the Integrin-N-cadherin binding which regulates migration of astrocytes and deposition of scarring tissues. This path upon activation these glias migrate into injured regions and generate scarring that prevents axonal regeneration. Integrin/N-cadherin-targeted therapies could modulate astrocyte function with a potential for reducing their migration as well as constraining scarring capacity since it has been shown that inhibition of Rho/ROCK, which is involved in cell shape regulation and motility could also play an important role for astrocytes (Zhu et al., 2025).

In animal models of SCI, several studies have shown that pharmacological manipulation of certain signal transduction pathways leads to functional recovery. By targeting the following pathways, where researchers are seeking environments which will encourage axon regeneration and recovery from loss of movement after injury. Such a treatment modality has been reported to improve endogenous regeneration processes and recovery of function following SCI (Schreiner et al., 2024).

Besides targeting astrocyte-related pathways, a potential treatment option in SCI is to target the immune response through modulating microglia activity. PD-1, which is also well-known for its immunoregulatory activity and has been suggested as an agent to promote the healing of injured spinal cords. It is expressed in activated T cells and also in microglia whose stimulation by this molecule inhibits the inflammation process and promotes the reparative response. It has been shown that modulating the activity of PD-1 can convert microglia into anti-inflammatory or reparative ones, thus decreasing the secondary injury and supporting neuronal cell protection. This shift in microglial phenotype is necessary to control the neuroinflammatory response that occurs after initial trauma. In preclinical models, administration of agonists and antagonists has resulted in improved outcomes. For example, using anti-PD-1 monoclonal antibodies or PD-1 ligand agonists, functional recovery was observed following spinal cord trauma in rodents. The studies further demonstrate that modulation of the microglia activity by targeting the PD-1 axis can contribute to neurorepair, as shown by preliminary studies in animals (Yuan et al., 2024).

Taken together, the combined inhibition of both Intergtin-N-cadherin axis and PD-1 pathway could be an effective treatment modality to treat SCI by inhibiting glial scarring as well as harmful inflammation. Thus, making the microenvironment more favorable to promote neuronal regeneration. These combinations of treatments might greatly enhance motor/sensory recovery following spinal cord injury, but further research is required to refine the therapeutic regimens and test their translatability into successful strategies for treating SCI.

### 4.8 Multi-target combination therapy

Multi-target combined treatment is a novel strategy to treat SCI, and which are complementary and boost each other’s neuroregenerative effects. This includes gene therapy, pharmacotherapy, physical therapy, or cellular therapy as potential therapeutic options to fight against such a complicated disease. It has been shown that the trans differentiation could be achieved by using genetic engineering tools like the forced expression of transcription factors such as NeuroD1 or Ascl1,could also promote the differentiation of astrocytes to functional neurons by triggering endogenous reparative mechanisms within the body (Liang et al., 2025), or cell-based therapy with umbilical cord-derived MSCs has shown promise for anti-inflammatory effects as well as tissue regeneration (Di et al., 2025). Such cells secrete exosomes, which contain bioactive molecules to communicate with other cells for wound healing purposes.

Studies have shown that successful cell–cell communication is vital for improved neuronal survival and protection (Wu et al., 2025). There are several therapies that have been explored, such as drugs for inflammation, oxidative stress, or glial scarring. Nevertheless, however, the success of these treatments are frequently confounded due to individual variation between patients and the complexity in which the pathology associated with SCI manifests itself (Di et al., 2025).

Successful application of all those different treatment strategies is an important factor for the best possible outcome. It has been shown, that combination therapy including stem cells and electrical stimulation may yield to significant improvement of motor recovery and anti-inflammatory effects in the animal model of SCI (Zhang Q. et al., 2025). Both of these treatment strategies have the potential to increase the electrical conductance through damaged nerve tissue, promote axon regrowth and prevent demyelination, showing the synergistic benefit for combining both intrinsic and extrinsic stimulation modalities (Zhang Q. et al., 2025). Secondly, a number of novel nanoformulations have recently been developed as biomimetic drug carriers using neutrophil membranes combined with exosomes derived from macrophages, providing an innovative way of transporting the therapeutics accurately to the lesions area for higher accuracy and efficiency in treatment (Shang et al., 2026).

Personalized therapies are an important component of this multifactorial treatment approach, as clinicians could improve the effectiveness of therapy and promote improved recovery by tailoring treatment to individual patients’ needs. Such personalized approaches include using outcome predictors to stratify patients, enabling determining when to administer a particular treatment, or what combination therapies should be administered in tandem with one another (Di et al., 2025). Personalized treatments could be further enhanced by integrating the analysis of all available multiomics data, which would also provide insight into the molecular- and cellular-level biology of the injured region, thus guiding selection of appropriate interventions (Di et al., 2025).

In conclusion, combination therapy with different targets is a new and promising approach for SCI treatment, suggests the need for a multimodal combined approach. This new strategy is based on synergies among gene therapy and cell replacement therapies, drug therapy, and physiotherapy with a great potential to achieve significant regeneration of the nervous system and improved outcomes after SCI. Further studies should aim at identifying optimal treatment orderings and cocktails as well as explaining underlying biological mechanisms of cooperation between them, and this is fueling innovation for SCI treatment.

### 4.9 Preclinical and clinical research progress

Preclinical studies have recently advanced the development of glial scar-targeted strategies to treat SCI, with preclinical studies in animals that have shown promising therapeutics effects. The role of the complicated neuroinflammatory response after SCI has been demonstrated in scientific researches; for example, abnormal activation of key signal transduction pathways such as NF-κB, NLRP3 inflammasome, and mTOR pathways might aggravate the inflammation response and promote hyper-glial scaring, thus impairing also neural repair (Darabniya, 2025). In experimental studies, targeting all three of these molecules together has been proposed to be neuroprotective, educe fibrosis, and/or improve recovery of movement. For instance, studies with selected pharmacological inhibitors and innovative biomaterial-based delivery systems have shown that they can positively modulate inflammation, thus allowing for the regeneration of the nerves and recovery in function (Nascimento et al., 2025). In addition, the use of stem-cell therapies (particularly those using MSCs) has been particularly promising.

MSCs were extensively studied in experimental animals that showed they can limit the formation of glial scars but at same time improve neuronal viability as well as promote axon regrowth (Lin et al., 2024). In particular, it has been emphasized that the combination of a suitable biomaterial and cell transplantation may lead to synergy between these two approaches, whereas these hybrid structures may provide both a physical scaffold and enhanced therapy of the implanted cells (Suzuki et al., 2023). All together, current lab research offers many new approaches that can overcome the difficulties of reactive gliosis, which paves the way toward treatment of human patients.

These agents are under investigation in early phase clinical trials for their toxicity profile and feasibility, and providing preliminary evidence of the potential to use them clinically in SCI. Much early work has focused on targeted drug delivery of small molecules which modulate specific inflammatory pathways, with the purpose of preventing scarring, while at the same time promoting axonal regrowth (Lu et al., 2025). For example, early-phase clinical trials of LLLT showed promising effects for modulating inflammation and promoting functional recovery after stroke (Mosleh et al., 2025). Early phase trials are important since these studies assess the safety and applicability to humans, thus laying the groundwork for future large scale clinical trials. In addition to studies on cell therapy methods such as OEC transplantation, has proven to be promising as preliminary results suggest that there may be benefits for scar tissue management and nerve fibre.

However, even if encouraging results have been obtained with animal models toward axonal regrowth, it is not straightforward to translate them into successful therapies for humans due to inter-individual differences or complexity of SCI pathology (Guha et al., 2025). In future clinical trials, the next step will be to optimize therapy including the schedule of administration, optimal dosages and optimal combinations in order to achieve better results for patients and address the multifactorial nature of SCI (Kalanchiam et al., 2025). Despite the challenges of taking these findings from bench to bedside, there is much promise in ongoing studies at the preclinical and clinical levels which may eventually lead to targeted therapies aimed at limiting gliosis and enhancing regeneration after SCI.

### 4.10 Challenges and future directions

**Figure 1:**
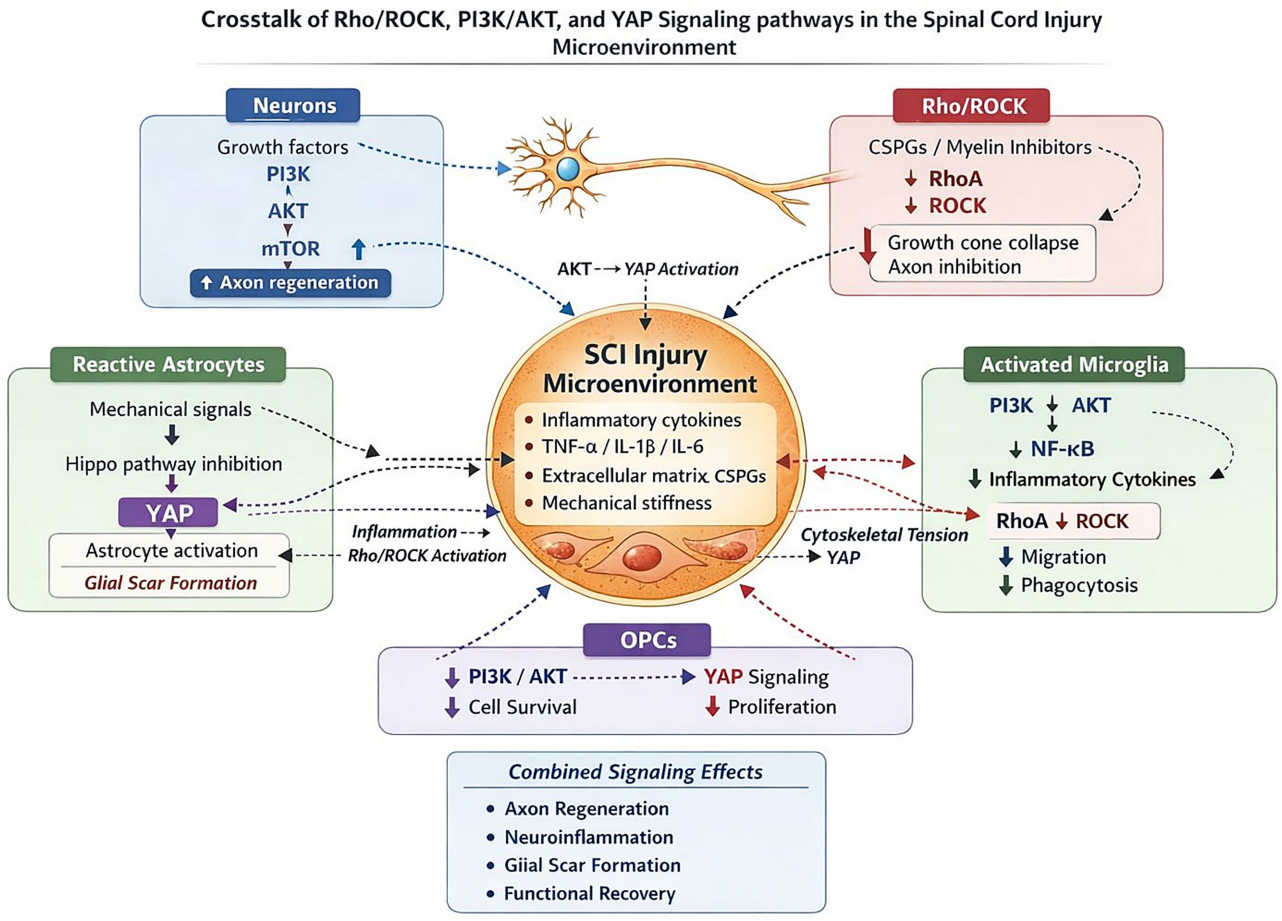
Signaling crosstalk regulating glial scar formation in the SCI microenvironment. In astrocytes, Rho/ROCK signaling regulates cytoskeletal remodeling, while YAP activation mediates mechanotransduction in response to ECM stiffness. Microglial NF-κB pathway drive inflammatory cytokine production. PI3K/AKT signaling exhibits phase-dependent effects, contributing to early neuroprotection but potentially promoting fibrosis during chronic stages. Crosstalk among inflammatory, mechanical, and proliferative pathways shapes scar architecture and regenerative potential.

SCI, which is difficult to treat due to the complicated mechanism including formation of glial scar with dual roles in SCI repair. The glial scar, which are mostly made up of reactive astrocytes, have both positive and negative properties: while they form a barrier to contain the inflammation and protect surrounding uninjured brain tissue against further damage. In contrast, they provide a poor environment which prevents axonal regeneration and repair (Yang et al., 2020), with such dual nature being difficult to manage by therapies aiming at modulating the scar in order to promote recovery. Current interventions often target scar inhibition, but maintaining such a shielding quality is clinically and scientifically challenging. Understanding precisely what biological mechanisms govern formation and degradation of a glial scar is essential to developing therapeutic strategies that modulate its formation but preserve protective roles. We concluded the therapeutic strategies according to the timing of intervention by displaying information (Table 1) and summarized these signaling pathways and their potential crosstalk within the injury microenvironment (Figure 1).

**Table 1:** Stage-specific therapeutic strategies targeting glial scar formation after spinal cord injury.

Injury phase	Dominant cellular events	Key signaling pathways	Pathological characteristics	Therapeutic goals	Representative strategies
Acute Phase (0–3 days)	Microglial activation; monocyte/macrophage infiltration; early astrocyte activation	NF-κB; HMGB1; p38 MAPK; early PI3K/AKT activation	Intense inflammation; cytokine surge; oxidative stress; blood–spinal cord barrier disruption	Limit secondary injury; suppress excessive inflammation; preserve neural tissue	Anti-inflammatory agents (e.g., HMGB1 inhibitors); PD-1 modulation; early MSC administration; NF-κB inhibitors
Subacute Phase (3 days–weeks)	Reactive astrogliosis; fibroblast migration; ECM deposition; macrophage polarization shift (M1 → M2)	Rho/ROCK; JAK/STAT; PI3K/AKT; TGF-β signaling	Astrocyte hypertrophy; CSPG secretion; early fibrotic scar formation	Modulate astrocyte phenotype (A1 → A2); control fibrosis; regulate ECM composition	ROCK inhibitors; gene regulation (e.g., Sox9 targeting); stem cell transplantation (MSCs, OECs); IL-4–based immunomodulation
Chronic Phase (weeks–months)	Mature glial scar stabilization; persistent astrocytes; ECM stiffening; CSPG accumulation	YAP signaling; Integrin–N-cadherin axis; mechanotransduction pathways	Dense fibrotic barrier; ECM rigidity; inhibition of axonal sprouting	Remodel scar architecture; enhance axon regeneration; improve neural plasticity	Chondroitinase ABC (ChABC); biomaterial scaffolds; micro-TENNs; rehabilitation (BWSTT); combinatorial multi-target therapy

Further insight into complex mechanisms at work in cell biology that underlie the formation of glial scars is necessary to develop more efficient treatments. Recent studies have highlighted important contributions from various cells, especially astrocytes, microglia and macrophages, play a key role in the regulation of inflammation and scarring following SCI (Singh et al., 2025). Understanding how this web of intermolecular signaling mediates such cell–cell interactions might lead to potential therapeutic strategies. In particular, recent studies indicate that manipulating macrophage polarization could be an effective way of changing this proinflammatory environment in order to create a more neuroprotective one (Hu and Gao, 2025). Furthermore, the development of extremely targeted drugs that can specifically inhibit negative components of scarring such as chondroitin sulfate proteoglycans, while retaining the protective functionality of scar tissue is a very important research avenue that has high clinical impact (Yu et al., 2025).

The recent developments in high-throughput single cell analysis techniques, gene editing, and nanotechnology may help to better understand the pathophysiology of SCI as well as develop innovative therapeutic strategies. Single-cell transcriptomics is able to identify and characterize single cell populations in the injured spinal cord, identifying new subpopulations of cells and inter-cellular interactions that could not be identified using traditional RNA-seq techniques (Cao et al., 2022). This fine-grained characterization can identify which subsets of cells drive scarring, as well as what role these cells play in wound repair. Furthermore, genome editing techniques like CRISPR-Cas9 provide means to precisely modify genes associated with scar tissue production and nerve cell regrowth, which could revolutionize medical therapies (Covache-Busuioc et al., 2025). For instance, in nanoscience, synthetic materials as well as drug delivery vehicles may offer new opportunities to more specifically deliver drugs to wound areas (Cheng et al., 2026).

A rational treatment of SCI will require overcoming these challenges associated with gliotic scar formation and thus requires an integrated understanding of the underlying biology combined with innovative therapeutic approaches. Further studies should focus in understanding of complex cell–cell communication and molecular mechanisms driving gliotic scar formation, although also reviewing the potential of new therapies utilizing novel technology. By combining these lines of investigation together, it is possible to lay a foundation toward more effective therapies which promote regeneration and recovery after SCI.

In short, glial scar formation after SCI is a double-edged sword that can be both protective and limiting in function. Although they protect neurons from further damage, they also block axonal regeneration and thus an optimal modulation of scarring is essential. It is a complex process involving many cells and signal transduction pathways suggesting that individualized therapies balancing neuroprotection and regeneration will be necessary in future. Potential strategies include gene therapy, transplantation cell therapy, and pharmacological treatment, together with physiotherapy, indicating that multi-modal treatment strategies should be considered. Further studies are warranted with a view toward individualized treatment for managing scar growth and minimizing damage to surrounding tissue, wherein translation between lab researches and clinics requires extensive trials and inventions. The understanding of clinic skills will be crucial to maximize the therapeutic effect of injured spinal tissues in real-life scenarios.
